# Myocilin Polymorphisms and Primary Open-Angle Glaucoma: A Systematic Review and Meta-Analysis

**DOI:** 10.1371/journal.pone.0046632

**Published:** 2012-09-28

**Authors:** Jin-Wei Cheng, Shi-Wei Cheng, Xiao-Ye Ma, Ji-Ping Cai, You Li, Guo-Cai Lu, Rui-Li Wei

**Affiliations:** 1 Department of Ophthalmology, Shanghai Changzheng Hospital, Second Military Medical University, Shanghai, China; 2 School of Life Sciences, Ludong University, Yantai, China; 3 Center for New Drug Evaluation, Institute of Basic Medical Science, Second Military Medical University, Shanghai, China; Vanderbilt University, United States of America

## Abstract

**Background:**

Glaucoma is the leading cause of irreversible blindness in the world. Recent evidence indicates a role for genetic susceptibility to primary open-angle glaucoma (POAG). The relation between myocilin polymorphisms and POAG susceptibility has been studied in different populations.

**Methods:**

A meta-analysis of 32 published genetic association case-control studies, which examined the relation between POAG and the R46X, R76K, Y347Y, T353I, and Q368X polymorphisms of the myocilin gene, was carried out.

**Results:**

In meta-analysis, significant associations were observed between POAG risk and two myocilin polymorphisms with summarized odds ratio of 4.68 (95%CI, 2.02–10.85) for Q368X and 2.17 (95% CI, 1.32–3.57) for T353I. Both Q368X and T353I were significantly associated with high-tension glaucoma, with summarized odds ratio of 4.26 (1.69, 10.73) and 2.26 (1.37–3.72). In Westerners, significant association was observed for Q368X mutation (odds ratio, 5.17; 95% CI, 2.16–12.40). However, in Asians it was for T353I (odds ratio, 2.17; 95% CI, 1.32–3.57).

**Conclusions:**

There is strong evidence that myocilin polymorphisms are associated with POAG susceptibility, and the prevalence of myocilin mutations might be ethnicity-dependent in Caucasians for Q368X and in Asians for T353I.

## Introduction

Glaucoma, which causes optic nerve damage and visual field loss, is the leading causes of irreversible blindness worldwide [1]. A family history of the disease has long been recognized as a major risk factor for glaucoma, suggesting that specific gene defects contribute to the pathogenesis of the disorder [2]. The most common form of glaucoma is primary open-angle glaucoma (POAG), which is characterized with typical optic disc damage and visual field defects, in an eye which does not have evidence of angle closure on gonioscopy, accompanied with elevated or normal intraocular pressure (IOP). Several chromosomal loci have now been reported as linked to POAG, such as myocilin (MYOC; GLC1A, MIM 601652), optineurin (OPTN; GLC1E, MIM 602432), and WD repeat domain 36 (WDR36; GLC1G, MIM 609669) [3].

The MYOC gene, also known as trabecular meshwork-inducible glucocorticoid response (TIGR) gene, was the first discovered to be linked to POAG in 1997 [4]. Several large studies have suggested that MYOC mutations are associated with 2% to 4% of POAG in patient populations worldwide, with more than 30 disease-associated mutations identified [5], [6]. The overall frequency of disease-causing mutations at MYOC is similar among African (4.44%), Caucasian (3.86%) and Asian (3.30%) probands with POAG [7]. Most disease-associated mutations at MYOC exist only in a specific racial group. The most frequent mutation Gln368Stop was present only in Caucasian descendants, and the second most frequent mutation Arg46Stop was shared only by Asian populations. However, the association of MYOC with POAG has been a source of controversy. After the initial discovery of POAG-causing mutations, the mutations were subsequently observed in controls, which were considered as non-disease-causing polymorphisms [8], [9]. Otherwise, reports published previously showed apparent non-consistent results. In familial studies, over four fifths Gln368Stop-carriers did not have POAG [10]. Also, the most frequent mutation Arg46Stop in Asians was even more often found in normal controls than in POAG probands [11]. Because the currently published studies only refer to a modest sample size, each one might not achieve a reliable conclusion. Hence, to investigate the association of the MYOC genetic variation with POAG susceptibility, a newly meta-analysis of all of the available case-control studies was carried out.

## Results

**Figure 1:**
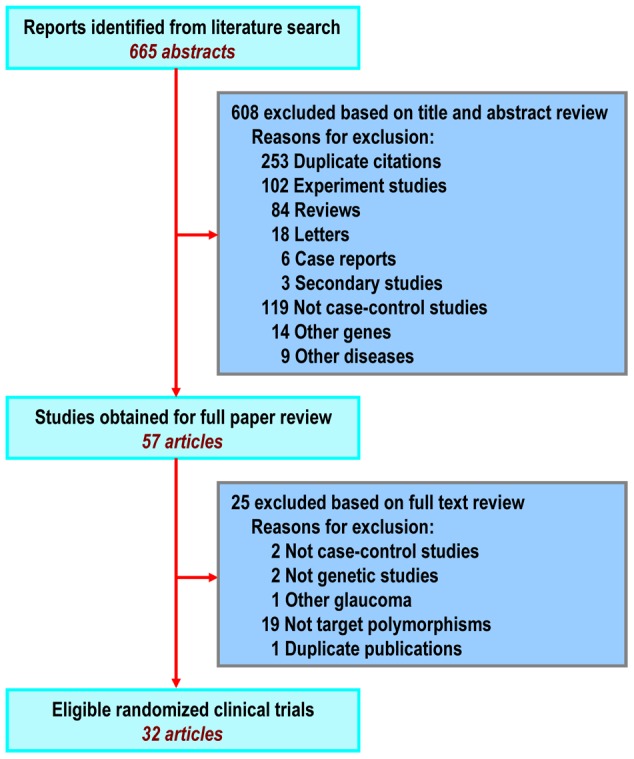
Flow diagram of study selection.

A total of 665 articles were identified across PubMed and Embase, and 57 full-text articles were retrieved. Finally, 32 studies met criteria and were included in the present meta-analysis [8], [9], [11]–[40]. The flow of study selection is shown in Figure 1, and the detailed characteristics of the studies were shown in Table 1. 6,729 patients and 4,871 controls were included in this study. Among those 32 included studies, 18 were conducted in Asians, 12 in Caucasians, and 1 in mixed. There were 19 studies for high-tension glaucoma (HTG), 1 study for normal-tension glaucoma (NTG), and 11 studies for both HTG and NTG. For R46X, R76K, Y347Y, T353I, and Q368X, meta-analyses were conducted within 11, 26, 11, 12, and 9 studies, respectively.

**Table 1:** Characteristics of publications included in meta-analysis of myocilin polymorphism and POAG.

Reference	Country	Ethnicity	Patients	Controls	No. (case/control)
Alward 1998	Iowa; Australia; US	Caucasian	POAG (HTG)	General population and healthy participants	716/596
Yoon 1999	Korea	Asian	POAG (HTG)	Non-glaucoma participants	45/106
Fingert 1999	Iowa; Australia; US; Canada; Japan	Caucasian; African; Asian	POAG (HTG)	General population and healthy participants	1693/793
Kubota 2000	Japan	Asian	POAG (HTG and NTG)	Non-glaucoma participants	140/100
Lam 2000	China	Asian	POAG (HTG)	Non-glaucoma participants	91/132
Vázquez 2000	Spain	Caucasian	POAG (HTG)	General population	79/90
Mabuchi 2001	Japan	Asian	POAG (HTG and NTG)	Non-glaucoma participants	233/100
Mataftsi 2001	Switzerland	Caucasian	POAG (HTG and NTG)	Non-glaucoma participants	117/50
Fan 2002	China	Asian	POAG (HTG)	Non-glaucoma participants	82/150
Faucher 2002	Canada	Caucasian	POAG (HTG)	General population and healthy participants	293/107
Hulsman 2002	Netherlands	Caucasian	POAG (HTG and NTG)	Non-glaucoma participants	50/100
Mukhopadhyay 2002	India	Asian	POAG (HTG)	Non-glaucoma participants	56/51
Pang 2002	China	Asian	POAG (HTG)	Non-glaucoma participants	201/388
Izumi 2003	Japan	Asian	POAG (NTG)	Non-glaucoma participants	80/100
Jansson 2003	Sweden	Caucasian	POAG (HTG)	Non-glaucoma participants	200/200
Melki 2003a	France	Caucasian	POAG (HTG and NTG)	Healthy participants	237/108
Melki 2003b	Morocco	Caucasian	POAG (HTG)	General population	57/60
Fan 2004a	China	Asian	POAG (HTG)	Non-glaucoma participants	157/155
Fan 2004b	China	Asian	POAG (HTG)	Non-glaucoma participants	32/96
Ishikawa 2004	Japan	Asian	POAG (HTG)	Healthy participants	171/100
Fan 2005	China	Asian	POAG (HTG and NTG)	Non-glaucoma participants	400/281
Rakhmanov 2005	Russia	Caucasian	POAG (HTG and NTG)	Non-glaucoma participants	170/100
Funayama 2006	Japan	Asian	POAG (HTG and NTG)	Non-glaucoma participants	532/240
Yao 2006	China	Asian	POAG (HTG and NTG)	Non-glaucoma participants	142/77
Bhattacharjee 2007	India	Asian	POAG (HTG)	General population and non-glaucoma participants	315/100
Kumar 2007	India	Asian	POAG (HTG and NTG)	Healthy participants	251/100
Lopez-Martinez 2007	Spain	Caucasian	POAG (HTG)	Healthy participants	110/98
Yen 2007	China	Asian	POAG (HTG)	Healthy participants	48/100
Bayat 2008	Iran	Caucasian	POAG (HTG)	Healthy participants	23/100
Jia 2009	China	Asian	POAG (HTG)	Non-glaucoma participants	176/200
Chen 2011	China	Asian	POAG (HTG)	Non-glaucoma participants	118/150
Whigham 2011	US	African	POAG (HTG and NTG)	Non-glaucoma participants	113/131

POAG: primary open angle glaucoma; HTG: high-tension glaucoma; NTG: normal-tension glaucoma.

**Figure 2:**
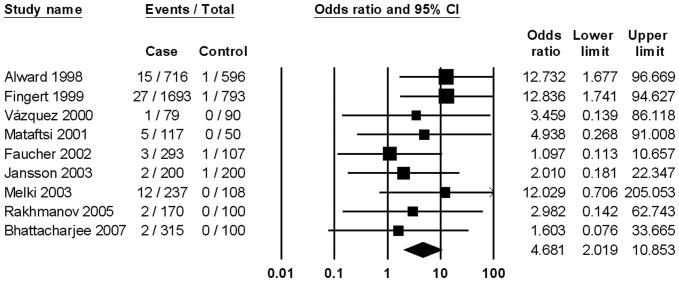
Meta-analysis of the association between primary open-angle glaucoma and myocilin Q368X mutation.

The association between the MYOC Q368X mutation and POAG was investigated with a total of 3,820 cases and 2,144 controls. Meta-analysis suggested that Q368X mutation carriage might be a risk factor for POAG with a summarized OR of 4.68 (95%CI, 2.02–10.85) (Figure 2), and no heterogeneity between studies (P = 0.76; I^2^ = 0.00%) was observed. There was no publication bias (P = 0.40 for Begg rank correlation analysis; P = 0.30 for Egger weighted regression analysis). In subgroup analysis by ethnicity, the association was significant in Caucasians, but not in Asians and Africans (Table 2). The association was also significant for HTG.

**Table 2:** Summary odds ratios from the meta-analysis of the association between primary open-angle glaucoma and myocilin polymorphisms.

Polymorphism and subgroup	No. of studies	Event/Total (%)		Odds ratios (95% CI)	Test for heterogeneity	Test for overall effect
		Case	Control			
**Q368X**						
All	9	69/3820 (1.8)	4/2144 (0.2)	4.68 (2.02, 10.85)	X^2^ = 4.974, P = 0.760, I^2^ = 0.00%	Z = 3.598, P = 0.000
Africans	1	1/312 (0.3)	0/90 (0.0)	0.87 (0.04, 21.58)	X^2^ = 0.000, P = 1.000, I^2^ = 0.00%	Z = −0.084, P = 0.933
Asians	1	2/315 (0.6)	0/100 (0.0)	1.60 (0.08, 33.67)	X^2^ = 0.000, P = 1.000, I^2^ = 0.00%	Z = 0.304, P = 0.761
Caucasians	8	66/3086 (2.1)	4/1905 (0.2)	5.17 (2.16, 12.40)	X^2^ = 4.563, P = 0.713, I^2^ = 0.00%	Z = 3.680, P = 0.000
HTG	7	52/3446 (1.5)	4/1986 (0.2)	4.26 (1.69, 10.73)	X^2^ = 4.467, P = 0.614, I^2^ = 0.00%	Z = 3.076, P = 0.002
**T353I**						
All	12	44/3452 (1.3)	25/2609 (1.0)	2.17 (1.32, 3.57)	X^2^ = 6.308, P = 0.852, I^2^ = 0.00%	Z = 3.041, P = 0.002
Asian	12	44/3452 (1.3)	25/2609 (1.0)	2.17 (1.32, 3.57)	X^2^ = 6.310, P = 0.852, I^2^ = 0.00%	Z = 3.040, P = 0.002
NTG	2	3/154 (1.9)	5/358 (1.5)	1.58 (0.40, 6.22)	X^2^ = 0.548, P = 0.459, I^2^ = 0.00%	Z = 0.656, P = 0.512
HTG	12	42/3298 (1.3)	25/2609 (1.0)	2.26 (1.37, 3.72)	X^2^ = 5.989, P = 0.874, I^2^ = 0.00%	Z = 3.176, P = 0.001
**Y347Y**						
All	11	174/3715 (4.7)	85/2164 (3.9)	1.20 (0.91, 1.57)	X^2^ = 3.719, P = 0.959, I^2^ = 0.00%	Z = 1.304, P = 0.192
Africans	2	8/425 (1.9)	2/221 (0.9)	1.37 (0.24, 7.88)	X^2^ = 0.857, P = 0.355, I^2^ = 0.00%	Z = 0.348, P = 0.728
Asians	2	9/457 (2.0)	0/177 (0.0)	3.24 (0.38, 27.46)	X^2^ = 0.308, P = 0.579, I^2^ = 0.00%	Z = 1.079, P = 0.281
Caucasians	8	157/2736 (5.7)	83/1717 (4.8)	1.19 (0.91, 1.57)	X^2^ = 2.057, P = 0.957, I^2^ = 0.00%	Z = 1.259, P = 0.208
NTG	2	3/68 (4.4)	7/177 (4.0)	1.89 (0.44, 8.23)	X^2^ = 0.421, P = 0.516, I^2^ = 0.00%	Z = 0.852, P = 0.394
HTG	6	157/3041 (5.2)	77/1747 (4.4)	1.22 (0.92, 1.63)	X^2^ = 2.522, P = 0.773, I^2^ = 0.00%	Z = 1.393, P = 0.164
**R76K**						
All	23	769/5371 (14.3)	608/3340 (18.2)	0.86 (0.69, 1.08)	X^2^ = 45.281, P = 0.002, I^2^ = 51.42%	Z = −1.319, P = 0.187
Africans	2	1/425 (0.2)	1/221 (0.5)	0.58 (0.06, 5.59)	X^2^ = 0.126, P = 0.723, I^2^ = 0.00%	Z = −0.473, P = 0.636
Asians	16	613/2999 (20.4)	461/2301 (20.0)	0.89 (0.75, 1.06)	X^2^ = 15.748, P = 0.399, I^2^ = 4.75%	Z = −1.339, P = 0.180
Caucasians	7	155/1947 (8.0)	146/800 (18.3)	0.62 (0.48, 0.81)	X^2^ = 44.682, P = 0.000, I^2^ = 86.57%	Z = −3.586, P = 0.000
NTG	5	64/625 (10.2)	85/798 (10.7)	1.19 (0.83, 1.73)	X^2^ = 4.586, P = 0.332, I^2^ = 12.78%	Z = 0.939, P = 0.348
HTG	17	559/4092 (13.7)	474/2701 (17.5)	0.84 (0.65, 1.08)	X^2^ = 37.401, P = 0.002, I^2^ = 57.22%	Z = −1.355, P = 0.175
**R46X**						
All	12	34/1826 (1.8)	35/1884 (1.9)	1.02 (0.61, 1.70)	X^2^ = 7.664, P = 0.743, I^2^ = 0.00%	Z = 0.073, P = 0.942
Asians	12	34/1826 (1.8)	35/1884 (1.9)	1.02 (0.61, 1.70)	X^2^ = 7.664, P = 0.743, I^2^ = 0.00%	Z = 0.073, P = 0.942
NTG	4	8/348 (2.3)	8/558 (1.4)	1.86 (0.60, 5.72)	X^2^ = 2.659, P = 0.447, I^2^ = 0.00%	Z = 1.080, P = 0.280
HTG	10	26/1359 (1.9)	35/1684 (2.1)	0.93 (0.55, 1.60)	X^2^ = 5.419, P = 0.796, I^2^ = 0.00%	Z = −0.250, P = 0.803

**Figure 3:**
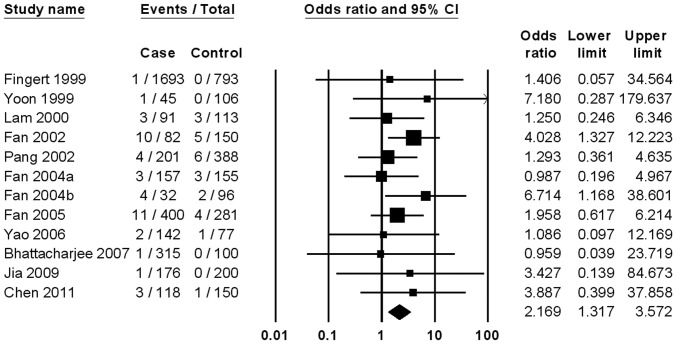
Meta-analysis of the association between primary open-angle glaucoma and myocilin T353I mutation.

It has been shown in Figure 3 that the T353I mutation was significantly associated with POAG (OR, 2.17; 95% CI, 1.32–3.57), with no evidence of heterogeneity among the overall 12 studies (P = 0.85; I^2^ = 0.00%). No publication bias was observed (P = 0.58 for Begg rank correlation analysis; P = 0.97 for Egger weighted regression analysis). Significant relation was also observed in Asians (Table 2). The ORs of T353I mutation were 2.26 (1.37–3.72) for HTG and 1.58 (0.40–6.22) for NTG.

Meta-analyses suggested that the other three MYOC polymorphisms Y347Y, R76K, and R46X were not associated with increased risk of POAG, with summarized ORs of 1.20 (0.91–1.57), 0.86 (0.69–1.08), and 1.02 (0.61–1.70) (Table 2). There was also no significant relation in the subgroup analyses by ethnicity or diagnosis criteria.

## Discussion

Myocilin is an eye protein found in the trabecular extracellular matrix, within the anatomic region that controls fluid flow, therefore, it might be the source of the resistance to aqueous humor outflow that causes the elevation of IOP in POAG [6]. Previous genome-wide analysis in a *Drosophila* ocular hypertension model identified transcripts with altered regulation and showed induction of the unfolded protein response upon overexpression of transgenic human glaucoma-associated myocilin [41]. This meta-analysis examined the MYOC Q368X, T353I, Y347Y, R76K, and R46X polymorphisms and their relationship to susceptibility to POAG. Thirty two articles addressing the most widely studied MYOC polymorphisms were identified, and their effects were summarized by means of meta-analysis. Significant associations with POAG susceptibility were observed for two loci mutations Q368X and T353I. Q368X variant carried near a 4.7-fold increased risk of POAG, and T353I variant carried near a 2.2-fold increase in POAG risk.

Strength of the associations in the subgroup analyses with respect to study population was not consistent. In Caucasians, significant association was observed for Q368X mutation, and, in Asians, it was for T353I. Because the rate of Q368X mutation was rare, of being 0.6% in Asians and 0.3% in Africans respectively [13], [33], the single study with small sample size might lead to the “negative” result in two populations. However, the absence of Q368X mutation was observed in other African [25], [40], [41] and Asian [13], [16], [18], [32] populations. Also, the T353I mutation was absent in Caucasian and African populations. Therefore, a different pattern of MYOC sequence variants might exist among different ethnic populations.

Normal tension glaucoma is a common form of open-angle glaucoma throughout the world, and yet there are many unanswered questions regarding both the mechanisms of the optic neuropathy. A certain level of IOP is the predominant causative risk factor in POAG, while additional IOP-independent factors take increasing importance in NTG [42]. It was proved that NTG was associated with mutations in OPTN [43]. However, a previous meta-analysis demonstrated no evidence of strong association between OPTN polymorphisms and susceptibility to HTG [44]. Therefore, both types of POAG might implicate different genetic susceptibility. In the present meta-analysis, both Q368X and T353I mutation of MYOC were generally shown to confer an increased risk of POAG with elevated IOP, but not for NTG. Although limited number of studies in the stratified analysis might explain, at least in part, the existing inconsistency. The disease-specific observation might suggest that there was different genetic susceptibility between HTG and NTG. Further studies addressing the association of MYOC on HTG and NTG, respectively, are warranted to verify the current findings.

Two previous meta-analyses have assessed the association between MYOC polymorphisms and POAG susceptibility [45], [46]. A previous meta-analysis, which based on 11 case-control studies, suggested an association of MYOC Q368X mutation and POAG [45]. Another meta-analysis of 4 case-control studies suggested that MYOC.mt1 polymorphism does not have significant influence on the risk of POAG development [46]. However, the previously published meta-analyses on the association of MOYC and POAG included the relatively less information and failed to confirm a strong and consistent association. The strength of present meta-analysis investigating the relationship between the MOYC polymorphic variant and susceptibility to POAG is based on the large amount of published data giving greater information.

Although we tried to conduct a thorough review of the existing literature, this study has several potential limitations. First, the possibility of selection biases cannot be completely excluded because all of the included studies were observational, and the potential confounding effect of age and sex might make the interpretation of the results and stratified analyses difficult. Second, only five POAG mutations were included in this analysis. Other potential polymorphisms, such as those at G12R, T123T, D208E, T285T, I288I, T325T, K398R, and A488A, were not included. Third, only published studies were included. Although multiple databases and websites were searched, unfortunately, it is possible that we may have failed to include some papers, especially those published in other languages. We can't find any evidence of publication bias by funnel plots, however, considerable between-study heterogeneity was found for R76K.

In conclusion, this systematic review summarized the strong evidence for an association between myocilin polymorphisms and POAG. Our results suggested Q368X and T353I variants of myocilin gene can be taken as reference loci for exploring POAG susceptibility, both in high-tension glaucoma. Furthermore, the prevalence of the two mutations of myocilin gene might be ethnicity-dependent, namely, in Caucasians for Q368X and in Asians for T353I.

## Methods

### Search Strategy

Studies addressing the association between MYOC mutations and polymorphisms and POAG were identified by searching for articles in the PubMed, and EMBASE until 31 December 2011. A broad search strategy combined terms related to gene (including keyword search using *myocilin*, *MYOC*, *trabecular meshwork-induced glucocorticoid response protein*, *TIGR*, *GLC1A*) and terms related to disease (including MeSH search using exp “*glaucoma, open angle*”, and keyword search using “*open angle glaucoma*” and its abbreviation). Additional studies were also identified by a hand search of all the references of retrieved articles.

We included only published manuscripts, without any language restriction. All the studies must meet the following inclusion criteria: (1) case-control study; (2) patients had to be POAG; and (3) Only the most widely mutations and polymorphisms were considered: R46X, R76K, Y347Y, T353I, Q368X. Exclusion criteria were: 1) studies with family-based designs; 2) studies on other polymorphisms other than the target polymorphisms.

### Data extraction

Data extraction was performed by two reviewers independently and in duplicate. For each study, the following data were extracted: first authors and publication year, country of origin, study base, study participant ethnicity, numbers of cases and controls, diagnosis criteria, demographic data, and genotype distributions for each polymorphism among cases and controls.

### Statistical Analysis

The association between MYOC polymorphism and POAG was calculated using co-dominant model. We used the odds ratio (OR) and corresponding 95% confidence intervals (CI) as the metric of choice. The statistical analysis was performed by Comprehensive Meta-Analysis (V2.0; Biostat, Englewood Cliffs, New Jersey, USA). The between-study heterogeneity was tested by the Q test and I^2^ test. If no heterogeneity detected (*P*>0.1), a fixed effects model was selected to pool the data. A random-effect model, otherwise, was employed after exploring the causes of heterogeneity. Stratified analyses were conducted with respect to ethnicity (Africans, Asians and Caucasians) and diagnosis criteria (NTG, HTG). Begg's rank correlation method and Egger's weighted regression method were used to statistically assess publication bias.
